# A Review: *Aedes*-Borne Arboviral Infections, Controls and *Wolbachia*-Based Strategies

**DOI:** 10.3390/vaccines9010032

**Published:** 2021-01-08

**Authors:** Samson T. Ogunlade, Michael T. Meehan, Adeshina I. Adekunle, Diana P. Rojas, Oyelola A. Adegboye, Emma S. McBryde

**Affiliations:** 1 Australian Institute of Tropical Health and Medicine, James Cook University, Townsville, QLD 4811, Australia; michael.meehan1@jcu.edu.au (M.T.M.); adeshina.adekunle@jcu.edu.au (A.I.A.); oyelola.adegboye@jcu.edu.au (O.A.A.); emma.mcbryde@jcu.edu.au (E.S.M.); 2 College of Medicine and Dentistry, James Cook University, Townsville, QLD 4811, Australia; 3 College of Public Health, Medical and Veterinary Sciences, James Cook University, Townsville, QLD 4811, Australia; diana.rojasalvarez@jcu.edu.au

**Keywords:** *Aedes*-borne, arboviruses, *Wolbachia*, vectors, controls

## Abstract

Arthropod-borne viruses (Arboviruses) continue to generate significant health and economic burdens for people living in endemic regions. Of these viruses, some of the most important (e.g., dengue, Zika, chikungunya, and yellow fever virus), are transmitted mainly by *Aedes* mosquitoes. Over the years, viral infection control has targeted vector population reduction and inhibition of arboviral replication and transmission. This control includes the vector control methods which are classified into chemical, environmental, and biological methods. Some of these control methods may be largely experimental (both field and laboratory investigations) or widely practised. Perceptively, one of the biological methods of vector control, in particular, *Wolbachia*-based control, shows a promising control strategy for eradicating *Aedes*-borne arboviruses. This can either be through the artificial introduction of *Wolbachia*, a naturally present bacterium that impedes viral growth in mosquitoes into heterologous *Aedes aegypti* mosquito vectors (vectors that are not natural hosts of *Wolbachia*) thereby limiting arboviral transmission or via *Aedes albopictus* mosquitoes, which naturally harbour *Wolbachia* infection. These strategies are potentially undermined by the tendency of mosquitoes to lose *Wolbachia* infection in unfavourable weather conditions (e.g., high temperature) and the inhibitory competitive dynamics among co-circulating *Wolbachia* strains. The main objective of this review was to critically appraise published articles on vector control strategies and specifically highlight the use of *Wolbachia*-based control to suppress vector population growth or disrupt viral transmission. We retrieved studies on the control strategies for arboviral transmissions via arthropod vectors and discussed the use of *Wolbachia* control strategies for eradicating arboviral diseases to identify literature gaps that will be instrumental in developing models to estimate the impact of these control strategies and, in essence, the use of different *Wolbachia* strains and features.

## 1. Introduction

**Figure 1:**
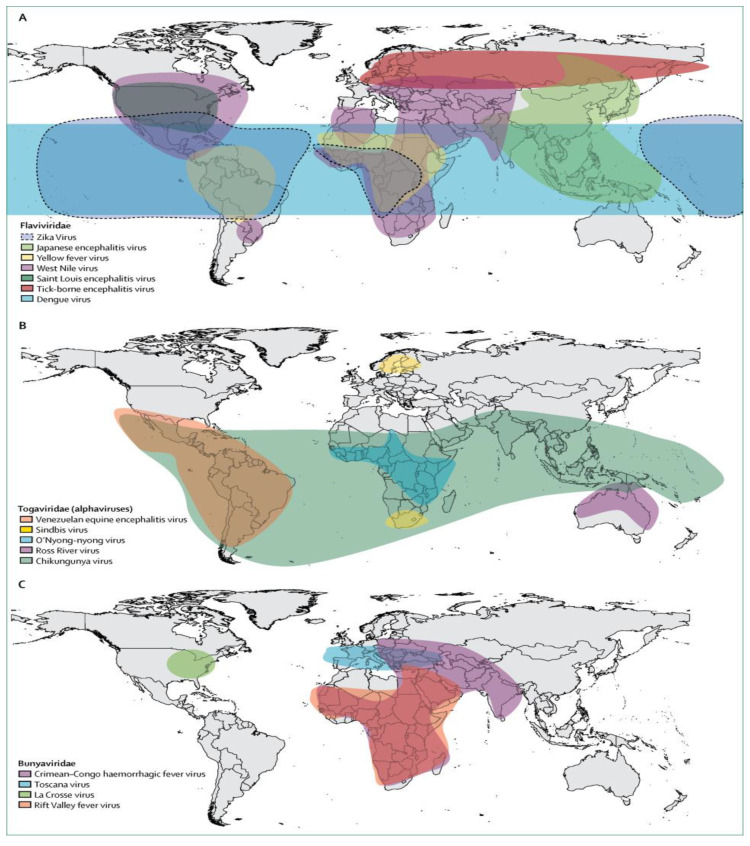
Global distribution of major arboviruses [7]. The *Aedes*-borne arboviruses described in this review belong to the *Flaviviridae* (ZIKV—light blue; YFV—yellow; DENV—blue) and *Togaviridiae* (CHIKV—green) families. Reproduced with permission from Elsevier; License no. 4892881108152.

Arboviruses (arthropod-borne viruses) are transmitted via blood feeding arthropods such as *Aedes* mosquitoes, flies, and ticks [1]. These viruses are characterised by either a double-stranded DNA or a RNA genome [2]. One and only example of the DNA genome of medical significance is the African swine fever virus (ASFV) which is mainly transmitted by ticks and belongs to the *Asfarviridae* family of viruses [3]. Except for the double-stranded DNA arboviruses, all other arboviruses have RNA genomes and are members of either of the *Flaviviridae, Togaviridae, Bunyaviridae, Rhabdoviridae*, and *Reoviridae* families [4]. Specifically, *Aedes*-borne viruses are a subset of arboviruses that are mostly transmitted by female *Aedes aegypti* mosquitoes and sometimes by female *Aedes* *albopictus* mosquitoes [5,6]. Examples of *Aedes*-borne arboviruses having RNA genomes are dengue virus (DENV), Zika virus (ZIKV), chikungunya virus (CHIKV), yellow fever virus (YFV), and Ross River virus (RRV) [7] (Figure 1). Other RNA arboviruses which are not *Aedes*-borne include West Nile virus (WNV) and Sindbis virus (*Culex*-borne) [8,9], Tick-borne encephalitis virus and Crimean-Congo haemorrhagic fever virus (tick-borne) [10,11], and Toscana virus (fly-borne) [12].

*Aedes*-borne viruses are fast spreading diseases that pose significant health problems globally [13,14,15] (Figure 1). These viruses can be life-threatening as dengue virus alone currently infects approximately 390 million people annually with 96 million of these showing clinical symptoms [16,17,18,19]. The global spread of these viruses is being fuelled by human migration, urbanization, and animal transportation [14,20,21]. Presently, there are no specific treatments for *Aedes*-borne infections [4,22]. However, supportive care for symptoms such as headache, seizure, and fever management and maintaining vital organs is available [2,20]. Additionally, some vaccines with high efficacy have been developed to prevent arboviral infections. They include 17D YFV [23], Japanese encephalitis [24] and tetravalent DENV vaccines [25]. However, research on the development of other arboviral infection vaccines such as ZIKV [26], WNV [27], RRV [28], and CHIKV [29] is still in progress but not yet approved. Full details of disease symptoms and available treatment alternatives are presented in Section 2.

To control the spread of *Aedes*-borne infections, several approaches such as those targeting human hosts, human-vector interactions and vectors specifically can be used [2]. Primarily, vector control strategies are used since they induce direct or biological reduction/elimination of the vectors without causing significant harm to human hosts [30]. The vector control strategies are classified into chemical, environmental, and biological control methods [2,30]. Presently, some of these methods are either widely practised or largely experimental (laboratory or field investigations).

Interestingly, one of the biological methods of vector control is the *Wolbachia*-based control method, which works by replacing existing wild-type mosquito vector populations with a *Wolbachia*-infected variant for which viral proliferation in its midgut is prohibited, rendering them less capable of transmitting the virus [31,32,33]. Several field studies have demonstrated the feasibility and effectiveness of *Wolbachia* introduction into native mosquito populations [34,35,36]. The main objective of this review is to critically appraise published literature on the available vector control methods and specifically highlight the use of the *Wolbachia*-based control method as a natural control measure for eradicating arboviral diseases. This includes both theoretical investigations of the potential efficacy of *Wolbachia*-based strategies and field trials that provide concrete demonstration. We also aim to identify literature gaps that will be instrumental in developing models to estimate the impact of this strategy. Therefore, we provide important background information on the types, scale, severity, and treatment of *Aedes*-borne arboviral infections, focusing on vector control methods and specifically highlighting those amenable to *Wolbachia*-type control.

## 2. *Aedes*-Borne Arboviruses

*Aedes*-transmitted arboviruses can be life-threatening when contracted by human hosts depending on the infection severity [37,38,39,40]. The primary vector responsible for the transmission of these arboviruses such as DENV, ZIKV, YFV, and CHIKV is the female *Aedes aegypti* (Yellowfever) mosquito, while female *Aedes albopictus* (Asian Tiger) mosquitoes also contribute to transmission [5,6]. DENV, in particular, is the most widespread *Flavivirus*, and also the most recognisable and deadly among the known *Aedes*-borne viruses [14] (Figure 1). Dengue viral infection can lead to health complications such as dengue haemorrhagic fever with shock syndrome and even cause circulatory failure and death [41]. The mean estimated intrinsic incubation period of dengue virus in humans is 5.9 days, while the estimated extrinsic (temperature-dependent) incubation period of the virus in the mosquito vectors is 15 and 6.5 days at 25 °C and 30 °C, respectively [42] (Table 1). In recent decades, the incidence of dengue viral infection has continued to increase. Modelling studies recently estimated that approximately 390 million dengue infections occur per year, with 96 million of these exhibiting clinical symptoms [16,17], and that the global population at risk of dengue is 3.9 billion [43].

Similar to DENV, ZIKV is transmitted through the infectious bite of *Aedes* mosquitoes. It was first isolated from a rhesus monkey in 1947 in an Ugandan forest: Zika [44]. Also, the vectors responsible for ZIKV transmission are *Ae. aegypti* and *Ae. albopictus* [45,46]. Although Zika viral infection is mainly transmitted via mosquito bites, instances of human-to-human and perinatal transmission have been observed [47,48,49,50]. There is evidence that ZIKV infection is associated with microcephaly, a congenital condition causing abnormal smallness of the head due to improper development of a baby’s brain during pregnancy or after childbirth [51]. Other symptoms of ZIKV are shown in Table 1.

Unlike DENV, a *Flavivirus*, chikungunya (meaning “to become contorted” in the Kimakonde language) virus: CHIKV is an *Alphavirus* that causes incapacitating joint pain and is transmitted by *Aedes* mosquitoes [52]. CHIKV transmission has also been reported through blood exposure [53]; infection of the human cornea [54]; and maternal transmissions—the latter of which can lead to miscarriage [55].

Furthermore, YFV is a member of the *Flaviviridae* family and is usually transmitted by *Aedes* mosquitoes [56]. The YFV infection can be severe, causing a high proportion of deaths in endemic populations [56]. YFV is a single-stranded RNA virus with a single serotype whose antigens are conserved [57]. The single serotypic nature of YFV allows the developed vaccine to protect the infected host against all the virus strains [58]. Human hosts are highly susceptible to contracting yellow fever infections as well as some non-human primates and rodents [59,60,61]. Recently, some studies have suggested that coinfection of arboviruses (Table 1) can not only occur, but can also generate cross-protective immunity where initial exposure to the first viral infection activates the immune response and confers acquired immunity against the next viral infection, and can also reduce the risk of subsequent infections for some arboviruses, in particular, dengue [62]. However, not all arboviral antibody responses are cross-protective as the interaction between some arboviruses and antiviral antibodies may result in a phenomenon known as antibody-dependent enhancement (ADE) of infection, which allows viruses to enter into the host cell [63]. This effect modulates the immune response of the host, facilitates viral production and may increase the severity of the viral disease [64]. 

## 3. Control Strategies for *Aedes*-Borne Viral Infections

**Figure 2:**
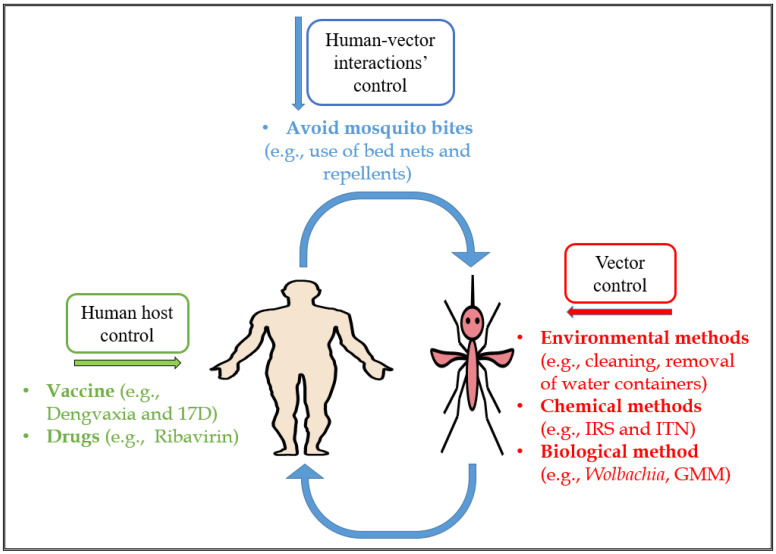
Diagram showing the summary of methods and examples (in brackets) that could be used to control *Aedes*-borne arboviral transmission: The green text refers to the human-host strategies, which could be used to prevent arboviral transmission via vaccines and drugs. The blue text refers to the human-vector interactions’ strategies that could be used for arboviral transmission control via humans avoiding bites from infectious mosquitoes. The red text refers to the vector control strategies that include chemical, environmental or biological methods to control arboviral transmission (IRS—indoor residual spraying; ITN—insecticide-treated bed nets; GMM—genetically modified mosquitoes).

*Aedes*-borne viral infection control has proven effective in reducing disease burden [94]. These strategies include taking preventive measures such as ensuring environmental cleanliness and adequate drainage, avoiding contact with vectors, vaccinating susceptible individuals and using genetic control of mosquitoes and paratransgenesis [41,95]. These measures can be grouped into three types of control measures depending on the stage of the transmission cycle that they target: (i) the human host; (ii) human-vector interactions; and (iii) vector control categories [2] (Figure 2).

Firstly, human host control strategies typically focus on reducing the susceptibility of humans to contracting *Aedes*-borne viral infections. This can be achieved through the use of vaccines and chemoprophylaxis (drug use) [23,24,25,96]. These control measures are used to inhibit, suppress or clear the virus, preventing replication in the human host [97]. Some vaccines with high efficacy have been developed, including the 17D yellow fever vaccine [23] and the Japanese encephalitis vaccine [24]. Notably, the tetravalent dengue vaccine has high protective efficacy rates of 56.5% and 60.8% against virologically-confirmed dengue but lower for DENV-2 [25]. A modelling study explored the third-year results of phase III trials of Dengvaxia and suggested that the vaccine generated protection against dengue within partially-immune persons but also increased hospitalizations among vaccine-sensitized individuals infected with dengue [66]. However, this vaccine is still controversial as it has been linked to significant side effects in the Philippines for instance [98]. Research on the development of vaccines for other *Aedes*-borne viral infections such as ZIKV [26], Ross-River virus (RRV) [28] and CHIKV [29] is still in progress.

Secondly, human-vector preventive measures prevent contact between susceptible human hosts and infected mosquitoes (and vice-versa), particularly mosquito bites. Examples include insecticide-treated bed nets, repellents [99] and sensitization of people in areas with high transmission rates to take preventive measures such as ensuring a clean environment and a good drainage system to avoid water stagnancy during rainfall [100]. Other preventive measures include the use of metofluthrin in the home as this has been shown to produce a rapid decrease in the observed biting frequency and increased kills among mosquitoes trapped inside the house [101]. Some studies in Australia and Vietnam have shown a significant reduction in mosquito population densities in homes treated with metofluthrin compared with those that were untreated [102].

Lastly, the vector control approach focuses on reducing the abundance, and inhibiting the transmission capacities, of virus-carrying mosquitoes [2]. Vector control can be challenging in endemic areas due to inadequate and haphazard implementation. Nevertheless, vector control techniques remain the main control strategies for suppressing dengue transmission, but often require a great deal of both financial and labour investment to achieve sustainability and sometimes do pose an environmental contamination risk, such as the use of chemical larvicides [30,103].

## 4. Vector Control Methods for *Aedes*-Borne Viral Infections

Vector control approaches are classified into three categories: environmental, chemical, and biological control methods [2]. Environmental methods include: cleaning of the environment, particularly, the mosquito vector breeding sites; covering or emptying water containers; and implementing strategic waste management schemes [30]. Chemical and biological control methods involve the use of insecticides such as Temephos and pyrethroids/organophosphates used in outdoor fogging [104]. Biological methods include the use of biological agents such as copepods, larvivorous fish, genetically modified mosquitoes and intracellular endosymbionts, e.g., *Wolbachia*, for control purposes [104,105,106]. Some of the environmental and chemical methods of vector control are widely practised while some biological methods are presently largely experimental.

### 4.1. Established Vector Control Methods

Environmental control methods for host vectors include common practices such as emptying, covering, or destroying water-filled containers, providing piped water, clearing and cleaning of the vectors’ breeding sites, and setting up strategies to ensure waste management implementation [30].

The chemical method generally involves use of a chemical mixture, solution or material to directly expel or, in most cases, kill arthropods such as mosquito vectors [107]. This method may be grouped into the use of: (a) durable treated materials such as door curtains and insecticide-treated bed nets (ITN); (b) insecticides for residual spraying, which include peri-domestic space treatments and indoor residual spraying (IRS) [108,109]; and (c) larval breeding control that includes the application of chemical larvicides such as Temephos to destroy breeding habitats [95,104]. Insecticides such as organophosphates and pyrethroids are most commonly used for the chemical control of *Aedes* mosqutoes [104]. However, there are limitations to chemical control methods. These limitations include environmental pollution, contamination, and toxicity [104], which may cause irritation to humans and endanger aquatic animal species.

Presently, chemical control methods are the most widely practised form of vector control, in particular the use of pyrethroids for outdoor fogging [110]. The direct killing of vectors using insecticides has been used for a long time and some studies have reported increased resistance to insecticides in mosquitoes, especially *Aedes aegypti* [108,109]. One of these studies investigated the insecticide resistance in *Aedes aegypti* mosquitoes in Ceara, Brazil and reported that these mosquitoes are subjected to selective pressure by the larvicide used, Temephos, as they reduce its effectiveness in the field. This resistance may be difficult to reverse as it may take more than seven years [108].

### 4.2. Experimental Vector Control Methods

Experimental vector control methods include the introduction of biological agents such as larvivorous fish [111], copepods (a group of small crustaceans) [112] or *Bacillus thuringiensis* [113], typically for larvae control. A study investigating the community effectiveness and efficacy of the use of larvivorous fish for dengue vector control reported that although the use of larvivorous fish could be effective in reducing the immature vector stages in small settings such as containers, these results could be minimal as it would require large coverage of multiple production of larvivorous fish containers to achieve any impact in an area of dengue endemicity [111]. Another similar study systematically reviewed the community effectiveness of copepods for dengue vector control in Vietnam [114]. The authors concluded that although there was an effective control of dengue transmission, the impact is difficult to determine as other control measures such as increased educational campaigns were combined with copepods [114]. A controlled study investigated the effectiveness of using *Bacillus thuringiensis israelensis* (BTI) spray to control the population of *Aedes* mosquitoes [113]. They showed that, although BTI treatment kills larvae, and thus suppresses adult mosquitoes indirectly, this effect is not sustainable over time [113]. Therefore, it can only be used together with other control measures as a supplement.

Other biological vector controls that are largely experimental at present may include both laboratory and field investigations. These investigations include the introduction of sterile insect techniques (SITs), genetically modified mosquitoes (GMM) and control agents that are incapable of transmitting viral pathogens [115], such as the *Wolbachia*-based strategy for disease control [31,116].

SITs are a method of insect control involving the rearing of large numbers of sterilized male mosquitoes that are released to mate with wild-type female mosquitoes resulting in the reduction of the reproductive advantage of the females [117]. This may lead to vector population suppression if sufficient releases of sterile male mosquitoes are rolled out. Sterilization can be achieved using radiation in dedicated facilities [118]. There are some drawbacks to SITs, which include difficulties in isolating male mosquitoes for sterilization and transportation problems, and overdose of radiation as this may also affect the physical strength of the sterilized mosquitoes [119,120]. The release of insects with dominant lethality (RIDL) which involves introducing a lethal trait into the female mosquitoes has emerged as a technique to overcome SITs difficulties [119,121]. A resulting example of the RIDL technique is the production of female flightless *Aedes* mosquitoes [122]. These flightless mosquitoes are created via RIDL using an indirect flight muscle gene *Act4* of the *Aedes aegypti* mosquito [122]. When this gene is switched on in developing female mosquitoes, it incapacitates the flight muscles leading to the death of the muscle cells and rendering the mosquitoes flightless [123]. This physical disability makes it difficult to fly, to find blood from human host, find a mate, and the mosquito easily becomes a prey for insectivores [123].

Studies have shown that genetic engineering can be used to modify the genetic features of mosquitoes to: resist viral infection; vaccinate humans against infection; and produce infertility in males [124]. However, studies describing ethical issues surrounding field trials of viral-resistant GMM deduced that for this technique to be rolled out, the disease of interest must pose a significant threat to public health in an area of isolation, as the greatest concern was for the protection of other community members who may be impacted but not enrolled in the study [125]. Additionally, the use of drives has interestingly increased the zeal for genetic control of mosquito vectors [126,127]. A study tested the first gene drives developed in *Aedes aegypti* mosquitoes. The authors confirmed that these drives, which are split so as to allow for drive safety performed excellently at very high frequency and also predicts that the split drives can be suitable for field trials to control local disease spread once the effectors are linked [126].

Another vector control technique that requires the introduction of a biological agent such as bacteria to control arthropod vectors is *Wolbachia*-based control [128,129]. Realistically, *Wolbachia*-based control is self-sustaining and the bacterium *Wolbachia* can be transmitted via transinfection to other insect species and is endosymbiotic in nature [130]. Although this strategy may require transinfection to successfully infect host vectors such as *Aedes* mosquitoes, it is not considered to be genetically modified because the *Wolbachia* bacterium is a natural endosymbiont that exists in most insect species [2].

## 5. *Wolbachia* Control Strategy

*Wolbachia* is an intracellular bacterium belonging to the *Anaplasmataceae* family [2]. This endosymbiotic bacterium naturally infects a wide range of invertebrate organisms such as arthropods and nematodes [130]. *Wolbachia* bacteria are found within the cytoplasm of the cells of their hosts, and they naturally exist in more than 50% of all insect species [131,132]. The *Wolbachia* endosymbiont is maternally (vertically) transmitted—the female *Wolbachia*-carrying arthropod passes the bacteria through the eggs to their offspring [133]. However, paternal (horizontal) transmission, which is very rare, has been observed in *Drosophila simulans* [134]. Paternal transmission can also occur under rare ecological circumstances such as the transmission of *Wolbachia* from infected to uninfected larvae of wasps sharing the same source of food [135]. While *Wolbachia* infection is not naturally present in all arbovirus-transmitting vectors such as *Aedes aegypti*, it can be introduced through stable transinfections of *Wolbachia* strains via microinjection [136]. The *Wolbachia* bacteria can be extracted from native hosts such as *Aedes albopictus* [137] and *Drosophila melanogaster* [133,138] and then injected into heterologous arthropods as with *Aedes aegypti*.

Most *Wolbachia* strains have derived their names from the host in which they were first discovered (Table 2). The first *Wolbachia* strain to be discovered was *w*Pip (*Wolbachia pipientis*) found in *Culex pipiens* mosquitoes [139]. Other strains include: *w*Mel found in *Drosophila melanogaster* (Fruit fly), *w*AlbA and *w*AlbB found in *Aedes albopictus* (Asian Tiger mosquito), and *w*Au found in *Drosophila simulans* [140]. The features of these *Wolbachia* strains may vary in their mosquito hosts due to high fitness cost and environmental factors such as high temperature (Table 2) [141,142,143].

The potential benefits of *Wolbachia*-based control techniques may be twofold: *Wolbachia* infection can disrupt arboviral replication and transmission; and the bacteria can also suppress vector populations [2,129,151].

### 5.1. Wolbachia-Based Disruption of Arboviral Transmission

The transinfection of *Aedes aegypti* with the endosymbiotic bacterium, *Wolbachia* could disrupt or inhibit arboviral transmission through four mechanisms [2]. The first is the competition for intracellular resource. Once present, *Wolbachia* bacteria can induce autophagy (cleaning or eating up damaged cells) in the arthropod’s cells [152]. To be able to survive, *Wolbachia* typically hijacks and regulates the autophagy system both within and outside the cell [153]. Similarly, arboviruses such as DENV and CHIKV rely on the autophagy system to replicate [154]. However, *Wolbachia* has the ability to manipulate the autophagy system set-up and interfere with some arboviral replications. This in turn, reduces the nutritional resources, such as cholesterol and iron, essential for viral growth [155]. Like *Wolbachia*, which is dependent on the arthropod cell cholesterol to multiply, *Aedes*-borne viruses such as DENV and CHIKV have been observed to manipulate the arthropod vector’s cell iron reserves [155]. In each event, both *Wolbachia* bacteria and arboviruses continually compete for limited host intracellular nutrients, resources, and space [156].

The second arboviral inhibitory mechanism is immune-priming. Immune-priming—also known as immune system preactivation—occurs when *Wolbachia* infection is transmitted into heterologous arthropods (i.e., non-native hosts of *Wolbachia* such as *Aedes aegypti*) via transinfection [157]. This mechanism preactivates the arthropod host immune system, which allows it to defend itself against arboviral pathogens [157]. According to a recent study, immune-priming can be induced by signalling pathways such as Immune deficiency (IMD), Toll and Janus kinase-signal transducer and activator of transcription (JAK-STAT) [2]. One study investigated the response of innate immune-priming in *Aedes aegypti* mosquitoes in the presence of *Wolbachia*-dengue interference [158]. It was shown that *Wolbachia* induced some immune genes involved in melanisation, Toll pathways genes and antimicrobial proteins such as peptides. The JAK-STAT pathway, which regulates the antiviral immunity and growth processes in arthropods has been shown to prevent DENV infection in *Aedes aegypti* mosquitoes [159]. An experimental study recently showed that immune-priming during the aquatic (larval) stage of *Aedes aegypti* mosquitoes with dormant DENV induced protection against the virus in the adult *Aedes* mosquitoes [160].

The third disruptive mechanism induces phenoloxidase (PO—an enzyme that increases the rate of phenol oxidation) cascade [161,162]. The importance of the PO cascade is that it produces melanin that accumulates around invading pathogens and at wound sites as this is known to have antipathogenic characteristics [162]. This cascade plays a critical role in the mosquito’s innate immune response to arboviruses. Studies have shown that *Wolbachia* bacteria increase melanization via the phenoloxidase activities in both homologous and heterologous arthropod vectors [161,162]. Therefore, a *Wolbachia*-induced phenoloxidase cascade may likely serve as protection against several arboviral infections [132].

The fourth mechanism is the miRNA-dependent immune pathway [163]. This pathway is an important component that modulates the arthropod hosts’ genes to control arboviral infection in many mosquito vectors [164]. Several miRNA-dependent immune responses and various metabolic processes needed for arboviral growth and replication are regulated in the presence of arboviral infections [165,166].

### 5.2. Wolbachia-Based Vector Population Suppression

The transinfection of *Wolbachia* into arthropod vectors such as *Aedes* mosquitoes may decrease their fitness, which in turn, leads to a reduction in the mosquito population [151]. One study previously reported that the introduction of a particular *Wolbachia* strain (*w*MelPop) into a mosquito could halve its life-span [167]. Another study conducted a survival experiment for three different *Wolbachia*-infected mosquito populations (*w*Mel, *w*AlbB, *w*MelPop) and wild-type mosquitoes stratified by sex (male and female). They showed that for the females, all *Wolbachia*-infected mosquitoes had significantly higher mortality rates compared with their wild-type counterparts. Similar results were observed for males, except for *w*Mel-infected mosquitoes whose lifespans did not differ significantly from the wild-type [151].

In practice, infecting *Aedes* mosquitoes with *Wolbachia* may also alter their reproductive lifecycle—potentially conferring *Wolbachia*-infected variants a competitive advantage over wild-type populations. One such mechanism is cytoplasmic incompatibility (CI) [129,168,169]. CI is a mechanism that induces incompatibility between the eggs and sperm of arthropods, in particular mosquitoes, enabling them to produce unviable offspring (no offspring) [170,171]. There are two types of CI: unidirectional and bidirectional CI. The former occurs when a *Wolbachia* infected male is crossed (mates) with an uninfected female mosquito (usually *Wolbachia* uninfected) and the resulting embryos are unable to mature into viable offspring [172,173]. However, the latter (bidirectional CI) describes the above inhibition mechanism but happening between crosses with infected mosquitoes with different strains of *Wolbachia* [174,175,176]. For example, the mating combination of a male and female mosquitoes infected with different *Wolbachia* strains are incompatible, thereby producing no viable offspring.

In general, the CI effect is not always dominant in all *Wolbachia*-infected arthropods as some *Wolbachia* strains do not exhibit this effect in some insect vectors. The CI effect may be fully present or absent depending on the *Wolbachia* strain and the arthropod host. For instance, in *Aedes* mosquitoes, studies have shown that the *Wolbachia* strains (*w*AlbA, *w*AlbB, *w*Mel) exhibit complete CI while *w*Au does not [141,144]. Several studies, in the case of mosquitoes, have shown that CI fuels the persistence of *Wolbachia*-infected mosquito populations and also confers a reproductive advantage on *Wolbachia*-infected female mosquitoes over the uninfected ones [128,129,177,178,179]. This persistence phenomenon in the presence of CI occurs because all mating patterns except crosses between uninfected male and female mosquito lines, produce *Wolbachia*-infected offspring [129,141].

Other features of *Wolbachia* infection that may suppress the vector population, include imperfect maternal transmission (IMT) [133,169,180], loss of *Wolbachia* infection (LWI) due to unbearable conditions (such as high temperature) [179], and superinfection of two strains of *Wolbachia* (which can occur in *Ae. albopictus* hosts) [181]. IMT rates may vary for different *Wolbachia* strains depending on some abiotic conditions such as altitude (higher IMT at high altitude compared to lower altitude) [182] and environmental factors (very low IMT under laboratory conditions but high IMT in the field) [183]. However, a particularly novel strain of *Wolbachia*: *w*Au, does not possess the CI feature [141]. Despite the non-induction of CI, *w*Au has been shown to produce high viral blockage and maintain *Wolbachia* infection at higher temperatures while other strains do not [141].

The effects created by the transinfection of *Wolbachia* in arthropods, which, in particular, resulted in viral blockage [141,144] and population reduction in arthropod vectors [31], make it a promising control strategy for the reduction and elimination of *Aedes*-borne infections [2].

## 6. Wolbachia-Based Field and Experimental Studies

Recent field studies have reported that *Wolbachia* can be used to suppress vector-borne disease transmission [36,129,132,133,138,141,184,185,186]. These studies showed that suppression can be achieved by introducing a *Wolbachia* strain into wild mosquito populations in the hopes of replacing the vector transmitting agent *Ae. aegypti* with one that is incapable of transmission [36,133,138,187]. The use of *Wolbachia* strains to control *Aedes*-borne viral infections such as dengue is categorized into three strategies: (a) introduction of *Wolbachia*-infected male mosquitos together with uninfected female mosquitoes causing CI [188]; (b) invasion of a strain of *Wolbachia* generating fitness reduction in an area of varying seasonality [167,189], e.g., by halving the life-span of mosquitoes after the introduction of a *Wolbachia* strain; and (c) invasion of a strain of *Wolbachia* that inhibits transmission by reducing the ability of the virus-carrying vectors to transmit infections [133,138,186,187]. These control strategies, which are not mutually exclusive, have reportedly been effective in Australia, Indonesia, Brazil, and Vietnam, leading policy makers, including The WHO, to encourage the use of these strategies in controlling the spread of *Aedes*-borne viral infections [129,190,191].

Previously, a study investigated the introduction *of w*AlbB *Wolbachia* strain into transgenic *Aedes aegypti* mosquitoes [192]. The study showed that the *w*AlbB infection in mosquitoes activates both IMD and Toll pathways and infection is maintained through maternal transmission (MT) [192]. Another study also showed that *Wolbachia* boosts immune responses and increase mosquitoes’ resistance to viruses, which allows the immune system to actively fight against the viruses in the arthropod host [193]. In a study series of blood-feeding mosquito trials in response to the human host, it was shown that as mosquitoes infected with *w*MelPop-*Wolbachia* strain age, they feed less compared to their uninfected counterparts as a result of the observed bent proboscis. This defect may cause tissue damage in mosquitoes as they age leading to a decreased bite rate [194]. One study [133] described the successful transinfection of *Aedes aegypti* mosquitoes with a *w*Mel-*Wolbachia* strain. It showed that this strain induces CI and high MT and also provides viral blockage of dengue serotype 2 infection transmission in *Aedes aegypti* mosquitoes.

Unlike other *Wolbachia* strains, the novel *w*Au strain displays some different characteristics in *Aedes* mosquitoes [141,144]. Some of the features include high retainment of *w*Au-*Wolbachia* infection at high temperatures and IMT [141]. In particular, this *Wolbachia* strain has been shown to be highly efficient in blocking arboviral transmission in *Aedes aegypti* [141] and *Aedes albopictus* [144] mosquitoes. However, the *w*Au strain does not induce CI [141,195] but may allow superinfection as bidirectional CI is ineffective in the presence of paternal transmission which itself is rare [134,141,144].

A study compared different *Wolbachia* strain features, such as high viral blockage and infection retention under high temperature, in transinfected *Aedes aegypti* mosquitoes [141]. The authors concluded that the *w*Au-*Wolbachia* strain was highly efficient in blocking DENV and ZIKV transmission and also provided more resilience to varying high temperature than the *w*Mel strain [141]. A similar study conducted in *Aedes albopictus* also showed that the special triple strain line (the generation of *w*AlbA-*w*AlbB-*w*Au line) created via *w*Au transinfection was completely resistant to arboviral infections like dengue and ZIKV [144]. Therefore, the *w*Au strain is a potentially promising control candidate as it maintains high frequency at high temperature and allows *Wolbachia* co-infection [141,144]. To support this reasoning, modelling the transmission dynamics between different *Wolbachia* strains possessing different features could contribute to the global reduction and elimination of *Aedes*-borne arboviral diseases.

## 7. Previous Studies on Mathematical Models of *Wolbachia*

In recent years, human and animal invasions of new ecosystems, environmental degradation, global warming, and downward economic trends such as financial recession have given rise to various types of arboviral diseases. These trends not only exacerbate infectious disease transmission, but also reduce access to efficient therapy due to poorer treatment retention or poorer living circumstances during recession periods [196]. In response to disease emergence, many researchers, epidemiologists in particular, have formulated and analysed mathematical models to understand the dynamics of disease transmission and to identify useful solutions [197,198,199,200].

In general, the introduction of mathematical models to understand the infection dynamics of diseases has long been helpful in the area of disease control [201]. One of the applicable concepts of mathematical models is computing the basic reproduction number (*R*_0_). *R*_0_ is the expected number of secondary cases produced, in a completely susceptible population, by a typical infective individual throughout his/her entire infectious lifetime. R_0_ can be used as a threshold to determine disease persistence (*R*_0_ > 1) or extinction (*R*_0_ < 1) [202].

In the context of arboviral control, mathematical models have been formulated to study the population dynamics of *Wolbachia*-infected mosquitoes invading naïve mosquito populations [180,203,204,205,206,207,208,209,210]. Some of these models introduced *Wolbacbia* strain(s) into a mosquito population and classified them into age-structured *Wolbachia*-infected and uninfected mosquito compartments [180,205,206,209]. These models were constructed to accommodate the population progression from offspring maturing to adult mosquitoes and reproducing; and examine the effects of IMT and CI which may determine the status and production of offspring respectively following the adult mosquitoes’ mating. A study by Ndii et al. [205] formulated a mathematical model for the *Wolbachia* interaction between the immature stages (aquatic stage), and the adult male and female mosquito populations to determine the persistence of mosquitoes infected with *Wolbachia* when competing with the uninfected ones. To do this, the authors derived the steady state solutions of the model and showed that maternal transmission, death, maturation, and reproductive rates determine the dominance of *Wolbachia*-infected mosquitoes. In a similar study, Xue et al. [209] considered the *Wolbachia*-induced fitness change and the CI effect. They showed that if the basic reproduction number is less than one, at which the disease typically dies out, an endemic *Wolbachia* infection can still occur if a sufficient number of the mosquitoes are introduced into the population. This is caused by backward bifurcation, where stable disease free and endemic equilibria co-exist [211].

Modelling investigations that estimate the impact of *Wolbachia* introductions in arboviral-endemic countries are surging by the day, as these studies tend to give insights into the appropriate time-dependent strategy in deploying *Wolbachia* as a means of controlling *Aedes*-borne infections [34,35,36]. In their study, O’Relly et al. [36] combined multiple modelling methods to first estimate the burden of dengue disease across separate jurisdictions in Indonesia, and then forecast the change in dengue prevalence following a wide-scale *Wolbachia* release program. They predicted a dramatic reduction in dengue transmission after a nationwide release of the *w*Mel *Wolbachia* strain. In particular, they estimated that there were approximately 7.8 million cases of symptomatic dengue in Indonesia in 2015 and attributed most of the gap in previous estimates of disease burden to underreporting (that is, asymptomatic and non-severe clinical cases that were challenging to diagnose in walk-in patients in hospitals or instances where patients did not go for treatment). The nationwide rollout of *Wolbachia* over the long term was estimated to avert 86.2% of these dengue cases [36].

A combined modelling-field study investigating the release of mosquitoes infected with the *w*AlbB strain was carried out in six different areas in Kuala Lumpur, the capital city of Malaysia [35]. The study showed that *w*AlbB-*Wolbachia* establishment was a success, maintained at high frequency in some sites and dominating at other sites following subsequent releases to overcome initial fluctuations.

Recently, one study modelled how the insecticide resistance of mosquitoes infected with *Wolbachia* could contribute to the local establishment of *Wolbachia* in a secluded area of Rio de Janeiro, Brazil, and validated the model results with experimental data [34]. After the release of two *Aedes aegypti* mosquito cohorts with different *Wolbachia* strains, *w*MelRio and *w*MelBr, the model clearly showed that *w*MelRio, which is resistant to pyrethroid pesticides, was able to establish while *w*MelBr, which is pyrethroid-susceptible, did not [34]. This implies that *Wolbachia*-infected mosquitoes resistant to pesticides may drive and establish *Wolbachia* infections in wild-type mosquito populations more readily than their pesticide-susceptible counterparts.

Another *Wolbachia* invasion model incorporated IMT and the loss of *Wolbachia* infection and showed that CI alone does not guarantee the establishment of *Wolbachia*-infected mosquitoes as IMT and *Wolbachia* loss could be more deleterious than CI is advantageous [180]. In effect, CI is not enough for *Wolbachia*-infected mosquitoes to dominate as both their intrinsic fitness and the possibility of mixed offspring play a critical role. Hence, we are interested in understanding how different features of *Wolbachia* infection, such as non-induction of CI, the high maintenance of the *Wolbachia* infection at high temperature, and the superinfection with different *Wolbachia* strains (*w*Au and *w*Mel) in mosquitoes could drive a reduction in arboviral transmission.

## 8. Literature Gap, Future Research, and Conclusion

*Aedes*-borne arboviral infections continue to be a public health problem globally [2,7,212,213,214,215]. Various control mechanisms for these viral infections are targeted at either suppressing the population of the virus-carrying vectors or inhibiting the viral replication in the vector hosts thereby hampering transmission [2]. Herein, we have typically described these control methods as: human host; human-vector interactions; and vector-focused (Figure 2). Of these control measures, the vector control methods, including environmental; chemical; and biological approaches, are the most widely used. Furthermore, this review highlights the importance of biological methods, specifically *Wolbachia*-based methods, in controlling *Aedes*-borne viral transmission.

The intracellular bacterium *Wolbachia* has been shown to reduce *Aedes*-borne viral infections such as DENV, ZIKV, CHIKV, and YFV in their endemic regions [129,133,138,141,145,186,194]. Although promising, the *Wolbachia* control strategy is not guaranteed to succeed as it faces the challenge of degrading potency at unfavourable weather conditions, among other limiting factors [179,216]. However, a novel *Wolbachia* strain, *w*Au, does not induce CI [141] yet is maintained even at high temperatures. This strain has been shown to produce high viral blockage, and induces stable superinfection when combined with other *Wolbachia* strains such as *w*AlbB in the vector host [141].

To better understand the dynamics of *Aedes*-borne viral infection both in human and vector hosts, there is a need to investigate the strategies of introducing *Wolbachia*-infected mosquitoes to control arboviral infection transmission. This can be done by formulating and analyzing mathematical models of different *Wolbachia* strains to capture the various important infection-driven features and validate these models using experimental data.

The research gaps identified in this review are: no modelling work on the combined three-vector control methods and no introduction of two *Wolbachia* strains with different characteristics such as the novel strain *w*Au-*Wolbachia* infected mosquitoes and its combination with other *Wolbachia* strains to quantify arboviral infection burden and control, have yet been performed. Therefore, in this review, we focus on the vector control methods together with different strains of *Wolbachia*-based control. Apart from greatly controlling virus proliferation in the midgut of *Wolbachia*-infected mosquitoes, the CI-absent *Wolbachia* strain, when subjected to high temperature is being retained in mosquitoes. This could be a successful strategy towards eliminating *Aedes*-borne infections. Hence, the need for in-depth insight and understanding of the different *Wolbachia* mosquito infection and superinfection dynamics and its impact when introduced into a mixed mosquito and human populations in arboviral endemic regions is sought in this regard.

Therefore, future work will include developing and comparing models for vector control methods incorporating the chemical, biological, and environmental control methods and comparing interventions. This would give great insights as it may require combining strategies such as outdoor fogging or use of chemical larvicides, educational campaigns to ensure clean drainages and covering of waterlogged containers, and sterile insect release or *Wolbachia*-infected mosquito rollout. In addition, the development of *Wolbachia* transmission models that describe the competitive dynamics between *Wolbachia*-infected and uninfected mosquitoes with different characteristics. It will also investigate the impact of releasing CI-absent *Wolbachia*-infected mosquitoes and its combination with other CI-present *Wolbachia*-infected mosquitoes in a human population infected with dengue and explore how single or combined strategies will impact on disease dynamics, in particular, the effectiveness of *Wolbachia* introduction in dengue endemic areas. These investigations will reveal the interactions between the different characteristics of *Wolbachia*-infected mosquitoes and dengue virus serotypes in the human host. These revelations will further contribute to the global effort to reduce or eliminate arboviral transmission.

## Figures and Tables

**Table 1:** *Aedes*-borne arboviral incubation periods and the asymptomatic fraction of infections

*Aedes*-Borne Arboviruses	Virus Type	Transmitted By	Symptoms	Supportive Treatment	Coinfection with Other Arboviruses	Intrinsic Incubation Period (Days)	Extrinsic Incubation Period (Days)	Asymptomatic Proportion in Infected Humans (%)
Dengue	*Flavivirus* [14]	*Ae. Aegypti* *Ae. albopictus* [5,6]	Sudden high grade fever, Headache, Nausea, Arthralgia, Eye and Muscle pain [65]	DENV vaccine and drug administration [66]	Yes (e.g., DENV and ZIKV) [67]	Median: 5.3 [68] Mean: 5.9 [42]	Mean: 15 (at 25 °C) 6.5 (at 30 °C) [42]	75 [69]
Zika	*Flavivirus* [70]	*Ae. aegypti* *Ae. albopictus* Human (via blood transfusion) [45,46,51]	Fever, Conjunctivitis, Muscle pain, Headache, Joint pain, Rash and Microcephaly [71,72]	Fluid intake and drug administration (such as acetaminophen) [73]	Yes (e.g., ZIKV and CHIKV) [74]	Median: 6.8 [75] 6.2 [76]	Median: 5.1 (at 30 °C) 9.6 (at 26 °C) 24.2 (at 21 °C) [77]	80 [78]
Chikungunya	*Alphavirus* [79]	*Ae. aegypti* *Ae. albopictus* [80]	High fever, Joint pain, Myalgia, Arthritis, Conjunctivitis, and Dermatologic manifestations [81,82]	Plenty of rest, Fluid intake and Acetaminophen [52,80]	Yes (e.g., CHIKV and DENV) [83]	Median: 3.0 [68]	Median: 2 [84]	Approx. 18 to 28 [85]
Yellow Fever	*Flavivirus* [56]	*Ae. aegypti* *Ae. Albopictus* [86]	Headache, Nausea, Vomiting, Fever, Dizziness and Joint pain [87,88]	YFV vaccine and Ribavirin [89,90]	Yes (e.g., YFV and CHIKV) [91]	Median: 4.3 [92] 4.4 [68]	Median: 10 (at 25 °C) [92]	55 [93]

**Table 2:** Description of different *Wolbachia* strains, the arthropod (origin) in which they were found and the presence of the means of control of arboviral transmissions

Wolbachia Strain	Origin	Means of Control of Arboviral Transmission (CI, MT, WIR, VB, F)	References
*w*Au	*Drosophila simulans/Fruit fly*	(No, Yes, High, High, Partial)	[141,144]
*w*Mel	*Drosophila melanogaster/Fruit fly*	(Yes, Yes, Low, Partial, Partial)	[31,133,145]
*w*AlbA	*Aedes albopictus/Asian Tiger mosquito*	(Yes, Yes, Medium, Partial, High)	[140,141]
*w*AlbB	*Aedes albopictus/Asian Tiger mosquito*	(Yes, Yes, Medium, High, Partial)	[129,141]
*w*MelPop	*Drosophila melanogaster/Fruit fly*	(Yes, Yes, Low, High, High)	[141,146,147]
*w*Pip	*Culex pipiens/Mosquito*	(Yes, Yes, -, Low, Low)	[129,148,149]
*w*Ri	*Drosophila simulans (Riverside)/Fly*	(Yes, Yes, -, Partial, Low)	[129,140]
*w*Inn	*Drosophila innubila/Vinegar fly*	(Yes [only males], Yes, -, Partial, Low)	[129,150]

CI—cytoplasmic incompatibility (Yes or No); MT—maternal transmission (Yes or No); WIR—*Wolbachia* infection retention (None, Low, Partial, High); VB—viral blockage (None, Low, Partial, High); and F—Fitness (None, Low, Partial, High). None—0, Low—<20%, medium—20%–90%, high—>90%.

## Data Availability

Data sharing not applicable.
